# Identification of Delivery Models for the Provision of Predictive Genetic Testing in Europe: Protocol for a Multicentre Qualitative Study and a Systematic Review of the Literature

**DOI:** 10.3389/fpubh.2017.00223

**Published:** 2017-08-22

**Authors:** Brigid Unim, Tyra Lagerberg, Erica Pitini, Corrado De Vito, Maria Rosaria Vacchio, Giovanna Adamo, Annalisa Rosso, Elvira D’Andrea, Carolina Marzuillo, Paolo Villari

**Affiliations:** ^1^Department of Public Health and Infectious Diseases, Sapienza University of Rome, Rome, Italy; ^2^Better Value HealthCare, Ltd., Oxford, United Kingdom

**Keywords:** genetic services, genetic delivery models, genetic testing, systematic review, expert interviews

## Abstract

**Introduction:**

The appropriate application of genomic technologies in healthcare is surrounded by many concerns. In particular, there is a lack of evidence on what constitutes an optimal genetic service delivery model, which depends on the type of genetic test and healthcare context considered. The present project aims to identify, classify, and evaluate delivery models for the provision of predictive genetic testing in Europe and in selected Anglophone extra-European countries (the USA, Canada, Australia, and New Zealand). It also sets out to survey the European public health community’s readiness to incorporate public health genomics into their practice.

**Materials and equipment:**

The project consists of (i) a systematic review of published literature and selected country websites, (ii) structured interviews with health experts on the genetic service delivery models in their respective countries, and (iii) a survey of European Public Health Association (EUPHA) members’ knowledge and attitudes toward genomics applications in clinical practice. The inclusion criteria for the systematic review are that articles be published in the period 2000–2015; be in English or Italian; and be from European countries or from Canada, the USA, Australia, or New Zealand. Additional policy documents will be retrieved from represented countries’ government-affiliated websites. The results of the research will be disseminated through the EUPHA network, the Italian Network for Genomics in Public Health (GENISAP), and seminars and workshops.

**Expected impact of the study on public health:**

The transfer of genomic technologies from research to clinical application is influenced not only by several factors inherent to research goals and delivery of healthcare but also by external and commercial interests that may cause the premature introduction of genetic tests in the public and private sectors. Furthermore, current genetic services are delivered without a standardized set of process and outcome measures, which makes the evaluation of healthcare services difficult. The present study will identify and classify delivery models and, subsequently, establish which are appropriate for the provision of predictive genetic testing in Europe by comparing sets of process and outcome measures. In this way, the study will provide a basis for future recommendations to decision makers involved in the financing, delivery, and consumption of genetic services.

## Introduction

The past decade has seen the emergence of Public Health Genomics (PHG)—a multidisciplinary field that has established scientific and policy foundations for the appropriate translation of genetic/genomics research into health benefits for individuals and populations (1). Still, many aspects of this nascent field need further investigation. This is particularly so as the rapid diffusion and extensive marketing of genetic tests for common diseases impact healthcare systems worldwide and raises questions on the proper provision of genetic services. A major concern regarding technology transfer in genetics is the premature introduction of genetic tests—i.e., the introduction of tests where the analytical and clinical validity, as well as the clinical utility, are not well documented. Although these issues are most concerning when considering predisposition testing, they may also apply to predictive testing. Another concern is the lack or insufficient evidence of cost-effectiveness of several genetic/genomic applications already introduced in clinical practice (2, 3). Particularly, there is a lack of evidence on what constitutes an optimal genetic service delivery model. This optimal model depends on the type of condition targeted and the healthcare context considered.

In order to add to the evidence base, the present project, therefore, aims to identify genetic service delivery models for the provision of predictive genetic testing in the European context, and to classify, and evaluate them. The genetic service delivery models will be compared between European and extra-European (Anglophone) countries (Canada, USA, Australia, or New Zealand). The project also aims to assess knowledge and attitudes of European public health (PH) professionals regarding the delivery of genetic services, and to obtain a picture of European PH community’s readiness to incorporate PHG into their practice.

For the scope of this project, predictive genetic testing (also known as pre-symptomatic testing) is defined as the use of genetic testing to predict whether an individual will develop a genetic disease at a later stage of their life; this term is only applicable where the disease-associated mutation is known and highly penetrant (4).

The study will be carried out within the Personalized pREvention of Chronic DIseases (PRECeDI) project (Marie Sklodowska-Curie Research and Innovation Staff Exchange 2014). The PRECeDI consortium is composed of five EU member countries (Italy, UK, Hungary, Spain, and the Netherlands) in collaboration with non-EU project partners (McGill University, Canada and Icahn School of Medicine at Mount Sinai, USA). The first task of the consortium was to obtain consensus among the project partners on the most suitable genetic tests that could be employed in the multicenter study. Four genetic tests have been selected during the preliminary meeting that took place in 2015 at Sapienza University of Rome (Italy). The selected genetic tests are for hereditary breast and ovarian cancer, Lynch syndrome, familial hypercholesterolemia (as examples of genetic tests of proven effectiveness and cost-effectiveness) (5), and familial thrombophilia (as an example of genetic test of unproven effectiveness and cost-effectiveness) (6, 7). The second task was to identify European countries that could participate in the multicentre study through consultation of researchers from the five-member countries of the PRECeDI consortium. The aim is to include as many countries as possible in order to obtain a more comprehensive picture of the provision of genetic testing and the implementation of genetic services delivery models in Europe.

**Figure 1:**
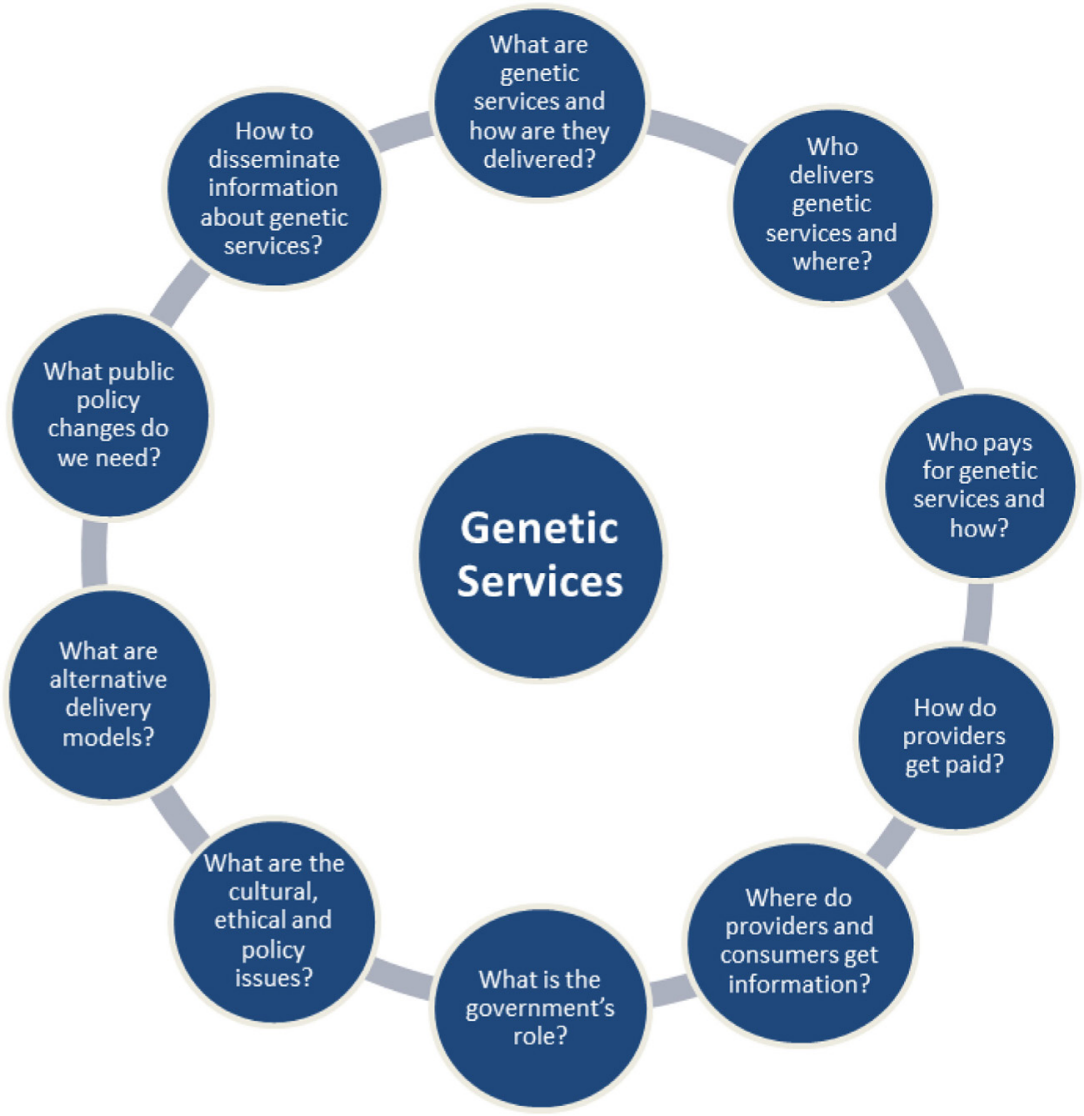
The 10 questions of the USA Genetic Service Policy Project Report (8).

There are a number of factors unique to each genetic service provision, which could be used to categorize genetic service delivery models. These factors have been summarized in 10 questions formulated by the USA Genetic Service Policy Project (GSPP) Report (8) (Figure 1) and include:

(i) practice setting and financial resources (public vs. private);
(ii) service provider and patient access [geneticists vs. primary care physicians/other medical specialists (e.g., cardiologists, oncologists, neurologists, endocrinologists, and so on)];
(iii) policy regulation (national and local policies, guidelines, protocols, and position statements);
(iv) laboratory practice standards (quality control standards, qualified personnel, etc.);
(v) information dissemination (methods of providing information about genetic services to patients and service providers).

The above factors reflect the three aspects of phase three (T3) of translation research in genomic medicine [where “translation” is defined as “the sequence of events in which a proven scientific discovery is successfully integrated into established practice and policy” (9)], namely integrating interventions into existing programs and structures (implementation research), promoting the adoption of the interventions by stakeholders (diffusion research), and increasing the spread of knowledge about evidence-based interventions (dissemination research).

The present project will, therefore, take all these factors related to genetic services into account in order to identify, classify, and evaluate genetic service delivery models for predictive genetic testing present in European countries and in selected extra-European (Anglophone) countries.

## Materials and Equipment

The project will be carried out through a multidimensional approach, which includes (i) a preliminary (non-systematic) literature search to define genetic services and genetic delivery models; (ii) a systematic review of published literature on existing genetic service delivery models and selected country websites for policy documents; (iii) structured interviews with health experts on genetic service delivery models, policies governing the use of genomics medicine, and evaluation of genetic testing and related services in their respective countries; and (iv) a survey of European Public Health Association (EUPHA) members’ knowledge and attitudes regarding the use of genomic applications in clinical practice.

### Stepwise Procedures

#### Preliminary Literature Search

Although the terms “genetic(s) service(s)” and “genetic delivery models” appear frequently in the peer-reviewed literature and in documents on genetics policy-related websites, they are usually not defined. Therefore, a preliminary (non-systematic) literature search was carried out to define the two terms. A common search strategy was used to identify articles from three electronic databases (Pubmed, Google, and Google scholar) with the following keywords: genetic(s) service, medical genetics, genetic service delivery, genetic(s) service delivery model, and genomic service delivery. Only articles defining the two terms “genetic(s) service(s)” and “genetic delivery models,” describing or classifying genetic service delivery models and published in English or Italian languages were included. No period restrictions were applied.

#### Systematic Review of the Literature

The systematic review focusing on existing genetic service delivery models will be performed according to the PRISMA Statement (10). The search will be conducted using five medical electronic databases (PubMed, Scopus, ISI Web of Knowledge, Google, and Google Scholar) with the following search term: genetic(s) services OR genetic(s) service provision OR genetic(s) service delivery OR genomic service delivery OR genetic(s) delivery models. The inclusion criteria will be:

– relevant articles and reports on pilot studies, best practices, and funded projects inherent to genetic service delivery;
– genetic services concerning the delivery of all types of genetic tests;
– genetic services for predictive genetic testing provided by genetic specialist teams, and healthcare professionals practicing in primary or secondary care;
– publication year between 2000 and 2015;
– studies published in English and Italian languages;
– interventions carried out in European and extra-European countries (the USA, Canada, Australia, and New Zealand).

The present project will focus on genetic service delivery models in Europe; therefore, other geographical areas considered will be used only for comparison.

The exclusion criteria will be:

– studies reporting only on genetic counseling services;
– descriptive studies where a specific delivery model cannot be identified;
– studies not specifying the type of genetic test offered.

Specific websites on PHG of EU countries (Table S1 in Supplementary Material) and extra-European countries (Table S2 in Supplementary Material) will be consulted as an additional resource to identify policy documents on delivery models (e.g., guidelines, protocols, position statements).

#### Experts’ Interviews on Genetic Service Delivery Models

Experts’ interviews will be conducted through an online questionnaire to:

(a) identify any genetic services delivery model that was not identified through the literature search;
(b) enhance the collection of process and outcome indicators used for quality assessment of genetic services delivery models;
(c) collect opinions of expert panels on the genetic services delivery models in their countries, in terms of strengths and weaknesses—that are also barriers and facilitating factors for the genetic model implementation—and possible improvements of the models.

Prior to the survey, a National Reference Group (NRG) will be established in each EU member state to select experts with different backgrounds and supervise the study at the national level. The panel of experts will have the following characteristics: (a) good knowledge or practical experience of at least one of the four types of selected genetic tests and (b) representative of each included country (e.g., in terms of regional or local autonomy in planning and delivery of health services, number, type, or geographic distribution of healthcare workforce in each country). The final number of experts will depend on the different contexts of each country.

The first part of the survey will focus on four types of genetic test (BRCA 1/2, Lynch syndrome, familial thrombophilia, and familial hypercholesterolemia). This part of the survey addresses healthcare professionals (e.g., medical geneticists, other medical specialists, and genetic counselors) working in genetic services with manager roles or in direct contact with patients requiring one of the four genetic tests. The NRG will select at least five experts for each genetic test to reach a minimum of 20 experts in each country. Stand-alone questionnaires are being developed for each genetic test with the following sections:

- Demographic and professional information;
- Genetic services delivery models for BRCA1/2, Lynch syndrome, familial thrombophilia, and familial hypercholesterolemia genetic testing.

The second part of the survey addresses health information management professionals who deal with health data collection and analysis at local (health facilities), regional (regional agencies), or national level (national institutes). The aim is to describe the flow and management of health information from each health facility to regional and national agencies where aggregate data are produced and used for planning activities. The NRG will select a minimum of five experts in each country. The related questionnaire will be composed of six sections:

- Demographic and professional information;
- Evaluation of genetic services activity;
- Evaluation of quality of genetic services;
- Evaluation of health outcomes;
- Electronic records and genetic information;
- Coverage of genetic services in the country.

The third part of the survey addresses experts in policy planning and policy research of genetic services employed in national institutes (e.g., National Health Institutes, ministries), universities or clinical research centers and it will focus on policy of genetic testing and related services. The NRG will select at least five experts in each European country. The questionnaire will be composed of the following sections:

- Demographic and professional information;
- Policy issues;
- Genetic services: access and availability;
- Professional education and training.

Once the selection process is completed, a link to the final version of the online questionnaire will be e-mailed to the participants. They will be given a time frame of 6 months to participate in the survey. Up to two reminder e-mails will be sent to non-responders 3 and 5 months after the initial e-mail to increase the response rate.

#### Knowledge and Attitudes of PH Professionals Regarding the Delivery of Genetic Services

Public health professionals may play different roles in the translation of genome-based knowledge and technologies into PH. They may use genomics tools to evaluate the health impact of PH interventions on different subsets of the population (1). Despite the fact that several surveys have been performed to evaluate knowledge, attitudes, and professional behaviors of physicians toward the integration of human genomic discoveries in clinical practice (11–16), only one study has been conducted for PH professionals. The study focused on knowledge, attitudes, and training needs of the members of the Italian Society of Hygiene, Preventive Medicine, and Public Health (S.It.I.) in the field of predictive genetic testing for chronic diseases (17). The study highlighted that Italian PH professionals have the required attitudinal background to contribute to the proper use of predictive genetic testing for chronic diseases, but need additional training to increase their methodological skills.

Looking ahead to the incorporation of PHG into PH practice in Europe, a similar survey will be conducted in a sample of European PH professionals, members of EUPHA. EUPHA is the umbrella organization for PH associations and institutes in Europe. It is composed of 71 member organizations from 41 countries and has about 5.900 individual members. The online survey will be carried out in order to obtain a picture of the European PH community readiness to incorporate PHG in their practice. The survey will focus on attitudes and knowledge of PH professionals toward genomic applications in clinical practice, the delivery of genetic services, evaluation of genetic service delivery models, and the role of PH professionals in the implementation of PHG. The questionnaire will be composed of the following five sections:

- Personal details;
- Professional activity;
- Knowledge of genetic testing and delivery of genetic services;
- Attitudes on genetic testing and the delivery of genetic services;
- Attitudes regarding the roles of PH professionals in PHG.

A link to the final version of the online questionnaire will be e-mailed to the 5.900 EUPHA members. The individual members could fit one of the following categories: (a) PH professionals involved in PHG; (b) PH professionals not involved in PHG; (c) not PH professionals involved in PHG (e.g., geneticists); (d) not PH professionals not involved in PHG (e.g., infectious diseases specialists). A filter question will direct professionals not involved in PHG activities to a reduced version of the questionnaire, comprised of only four items in sections C and D.

Participants will be given a time frame of 6 months to participate in the survey. To increase the response rate, up to two reminder e-mails will be sent to non-responders 3 and 5 months after the initial e-mail. In case the response rate should not be sufficient, hard copies of the study will also be distributed to the participants during a EUPHA Conference. A pilot phase will be conducted prior to administration of the questionnaire to EUPHA members in order to ensure practicability, validity, and correct interpretation of results.

#### Data Management

Data from the literature review and surveys will be collected and analyzed by the authors. A data extraction form has been developed specifically to collect relevant information from the studies included in the systematic review (Table S3 in Supplementary Material) and is composed of three parts:

– General description of the study and the genetic service. This section collects general information about the study (i.e., authors, title of the study, etc.) and the genetic service (i.e., practice setting, financing mechanism, etc.);
– Information on patients and pathways to care. This section investigates the characteristics of the target population of the genetic service and pathways to care, as well as cost-effectiveness and efficacy of the genetic service;
– Genetic service evaluation. This section investigates the type of genetic service delivery model, strengths, and weaknesses of the model, as well as the genetic service capacity in terms of population and geographic area served, staff qualification, laboratory characteristics, and outcome evaluation.

Five members of the working group made an independent evaluation of each genetic service using the data extraction form, followed by extensive group discussions. Eventual discrepancies were resolved after discussion with the coordinators of the project. The coordinators were responsible for revising and standardizing the preliminary results and for supporting the working group throughout the whole evaluation process.

In the experts’ interview, the diffusion of each genetic service delivery model in each country will be assessed through a three-point Likert scale (“poorly diffused,” “sufficiently diffused,” “highly diffused”), while the likelihood that a specific genetic services pathway is associated with one of the identified delivery models will be assessed by another three-point Likert scale (“unlikely,” “likely,” “very likely”).

In the EUPHA survey, attitudes of PH professionals will be assessed through a three-point Likert scale (“agree,” “uncertain,” and “disagree”), while knowledge will be assessed through a combination of multiple choice questions and three-point Likert scale answers.

Internal consistency of all questionnaires will be assessed by obtaining Cronbach’s alpha coefficients.

The final results of the project will be available in 2019 and will be disseminated through the EUPHA network, the Italian Network for Genomics in Public Health (GENISAP), seminars and workshops. In particular, internal workshops and open seminars have been scheduled for the period 2016–2018.

## Anticipated Results

The preliminary literature search produced eight records useful in defining the terms “genetic(s) service(s)” and “genetic delivery models” and in identifying the wide range of genetic services provision (8, 18–25). The definitions of genetic services were provided by the GSPP (8) (“genetic testing, diagnosis of genetic conditions, genetic counseling, and treatments for individuals with or at risk of genetic disorders”) and the article by Silvey et al. (18) (“medical genetic services are provided by specialist genetic centers and they include activities such as diagnostic laboratory services, education of healthcare professionals, participation in research, and expert advice to policy makers”). The two definitions of genetic services are comprised in the Italian “Guidelines for Medical Genetics Activities” (19) in which genetic services are defined as specialized services offered to individuals and families with genetic conditions or at risk of developing or transmitting a genetic condition. The guidelines also state that genetics services comprise clinical genetics (genetic counseling, diagnosis, treatment, and follow-up) and genetic laboratory services (genetic testing). Genetics services collaborate in education and training of healthcare professionals and provide information to the public. The definition of genetic services reported by the Italian “Guidelines for Medical Genetics Activities” has been adopted by the working group of the present project.

Particularly useful in defining the term “genetic delivery models” were the 10 questions of the GSPP Report (8), which summarize the main aspects inherent to the delivery of genetic services (Figure 1). For the purpose of the present project, a genetic service delivery model for the provision of predictive genetic testing is defined as the *broad context within the* PHG *framework in which genetic services are offered to individuals and families with or at risk of genetic disorders*. In other words, a genetic delivery model is a combination of personal healthcare services provided by healthcare professionals to individuals and families (i.e., diagnosis, treatment/management, and information) and PH services and functions (i.e., population screening, financing, policy development, workforce education, information/citizen empowerment, service evaluation, and research).

The two articles by Gu et al. (22, 23) and the review by Battista et al. (25) provided a classification of genetic service delivery models. Battista et al. (25) classified genetic service delivery models into four types:

(i) multidisciplinary specialist clinics and coordinated services in rare genetic disorders led by geneticists;
(ii) genetic services integrated in other medical specialties (e.g., oncogenetics, neurogenetics, cardiogenetics);
(iii) genetic services integrated into primary care;
(iv) genetic services provided in screening programs (e.g., prenatal and newborn screening).

The classification by Battista et al. (25) centers on the role of the healthcare professional involved in the provision of genetic services in each model (i.e., making referrals to genetic services for genetic counseling and/or genetic testing, direct request of a genetic test, interpretation of results, etc.). The article states that genetic services were provided mainly by geneticists, but in recent years more specialists are involved in genetic service provision alone or in collaboration with geneticists (e.g., primary care physicians/other specialists can carry out risk assessment, counseling and request genetic testing and interpret the results or refer patients to genetic services after risk assessment).

The classification provided by Gu et al. (22, 23) instead focuses mainly on the patients’ pathway from the point of access to the genetic service to diagnosis and treatment of the genetic disorder. The articles describe patients’ pathways prior to and after genetic testing, and include the commercial model where a genetic test can be ordered directly by an individual without involving healthcare professionals:

(i) The Patient–Doctor–Counselor Model;
(ii) The Patient–Doctor–Lab Model;
(iii) The Patient–Counselor–Lab Model; and
(iv) The Patient–Lab (Commercial) Model (i.e., direct-to-consumer genetic testing).

The final classification of genetic services delivery models will take into consideration the delivery models described by Battista et al. (25) and all possible patients’ pathways described by Gu et al. (22, 23) or identified through the systematic review of the literature. Each genetic service provision identified will be described and compared between European countries and in selected extra-European countries through a set of factors that have been summarized by the USA Report (GSPP; see Figure 1) (8).

The process and outcome indicators collected from the present project will be used to define a minimum set of indicators necessary for the assessment of genetic services through a consensus procedure among the partners of the PRECeDI project (Italy, UK, Hungary, Spain, the Netherlands, Canada, and the USA).

With regard to knowledge and attitudes of PH professional, we can anticipate that poor knowledge of genetics will be a barrier to the provision of genetic services (e.g., risk assessment, counseling, interpretation of genetic test results, and so on). It will surely interfere with the process of incorporating genetics into clinical practice.

Results of this project may be disseminated in scholarly articles and at academic conferences but no personal information about the participants will be used in the reports or manuscripts.

## Discussion

The transfer of genomic technologies from research to clinical application is influenced not only by several factors inherent to research goals and delivery of healthcare but also by external and commercial interests that may cause the premature introduction of genetic tests in the public or private sector (i.e., introduction of a test despite insufficient evidence regarding its analytical validity, clinical validity, and utility). Furthermore, current genetic services are delivered without a standardized set of process and outcome measures, which are essential for the evaluation of healthcare services. It is important that only genetic/genomic applications with proven efficacy and effectiveness are delivered to populations, and particularly that technologies have favorable cost-effectiveness ratios (21). This study will facilitate the identification of appropriate models for the provision of predictive genetic testing in the European context by identifying, classifying, and subsequently comparing outcome and process measures of genetic service delivery models. Such classification and consideration of process and outcome indicators will facilitate the comparison of models across different countries and health systems. The EUPHA survey will be useful to describe and compare current points of views, information, and educational needs of PH professionals in Europe. Health professionals’ knowledge and attitudes regarding the delivery of genetic services is an indicator of the European PH community’s preparedness to incorporate PHG into their practice. Hence, this indicator could be a barrier or a facilitating factor for the implementation and provision of genetic services in Europe.

In conclusion, the current project will identify possible points of improvement for currently implemented genetic services delivery models in Europe and provide recommendations to decision makers involved in the financing, delivery, and consumption of genetic services.

## Ethics Statement

The project is carried out in accordance with the Italian data protection code for sensitive data (Legislative Decree no. 196/2003) and informed consent will be obtained through an online consent form in accordance with the Declaration of Helsinki. The protocol has been submitted to the Ethics Committee of Sapienza University of Rome, Italy.

## Author Contributions

All authors contributed to the conception and design, and are currently carrying out the study. BU, TL, and PV wrote the present manuscript. All authors read and approved the final manuscript.

## Conflict of Interest Statement

The authors declare that the research was conducted in the absence of any commercial or financial relationships that could be construed as a potential conflict of interest.

## Abbreviations

PHG, Public Health Genomics; EUPHA, European Public Health Association; NRG, National Reference Group; GENISAP, Italian Network for Genomics in Public Health; GSPP, USA Genetic Service Policy Project; PRECeDI, Personalized pREvention of Chronic DIseases.
