# Supplementary material for: Identification of Delivery Models for the Provision of Predictive Genetic Testing in Europe: Protocol for a Multicentre Qualitative Study and a Systematic Review of the Literature
Source: Front Public Health. 2017 Aug 22;5:223. doi: 10.3389/fpubh.2017.00223 (PMC5572240; doi:10.3389/fpubh.2017.00223)
Supplement: Supplementary file 1 [file table_1.docx]

Table 1 - WEBSITES CONSULTED AS A STARTING POINT FOR IDENTIFICATION OF POLICY DOCUMENTS IN EU COUNTRIES

| COUNTRY | **ORGANIZATION** | **WEBSITE** |
| --- | --- | --- |
| **Austria** | Federal Ministry of Health | http://www.bmg.gv.at/home/EN/Home |
| **Belgium** | Health, Food Chain Safety and Environment | http://www.health.belgium.be/en |
| **Bulgaria** | Ministry of Health | http://www.mh.government.bg/bg/ |
| **Croatia** | Ministry of Health | https://zdravlje.gov.hr/ |
| **Cyprus** | Ministry of Health | http://www.moh.gov.cy/moh/moh.nsf/index_en/index_en |
| **Czech Republic** | Ministry of Health of the Czech Republic | http://www.mzcr.cz/En/ |
| **Denmark** | Ministry of Health | http://www.sum.dk/English.aspx |
| **Estonia** | Ministry of Social Affairs | https://www.sm.ee/en |
| **Finland** | Ministry of Social Affairs and Health | http://stm.fi/en/frontpage |
| **France** | Ministry of Social Affairs and Health (Ministère des Affaires Sociales et de la Santé) | http://social-sante.gouv.fr/ |
| **Germany** | Federal Ministry of Health | http://www.bmg.bund.de/en.html |
| **Greece** | Ministry of Health and Social Solidarity | http://www.ermis.gov.gr/portal/page/portal/ermis/publicBodies?p_topic=8500 |
| **Hungary** | Ministry of Human Capacities | http://www.kormany.hu/en/ministry-of-human-resources |
|  | Research Centre for Molecular Medicine | http://rcmm.med.unideb.hu/ |
|  | Hungarian Academy of Sciences | http://www.szbk.u-szeged.hu/ |
| **Ireland** | Department of Health | http://health.gov.ie/ |
| **Italy** | Ministry of Health | http://www.salute.gov.it/ |
|  | SIGU (Società Italiana di Genetica Umana) | http://www.sigu.net |
| **Latvia** | Ministry of Health of the Republic of Latvia | http://www.vm.gov.lv/en/ |
| **Lithuania** | Ministry of Health of the Republic of Lithuania | https://sam.lrv.lt/en/ |
| **Luxembourg** | Ministry of Health (Health Portal website) | http://www.sante.public.lu/fr/index.php |
| **Malta** | Ministry of Health | http://health.gov.mt/en/Pages/health.aspx |
| **The Netherlands** | Ministry of Health, Welfare and Sport | https://www.government.nl/ministries/ministry-of-health-welfare-and-sport |
| **Poland** | Ministry of Health | http://www.mz.gov.pl/en/ |
| **Portugal** | Directorate General of Health | http://www.dgs.pt/directorate-general-of-health.aspx |
| **Romania** | Ministry of Health | http://www.ms.ro/ |
| **Slovakia** | Public Health Authority of the Slovak Republic | http://www.uvzsr.sk/en/ |
|  | Ministry of Health of the Slovak Republic | http://www.health.gov.sk/Index.aspx |
| **Slovenia** | Ministry of Health | http://www.mz.gov.si/en/ |
| **Spain** | Ministry of Health, Social Services, and Equality | http://www.msssi.gob.es/en/home.htm |
| **Sweden** | Ministry of Health and Social Affairs | http://www.government.se/government-of-sweden/ministry-of-health-and-social-affairs/ |
|  | National Board of Health and Welfare (Socialstyrelsen) | http://www.socialstyrelsen.se/nationalguidelines |
|  | Public Health Agency (Folkhalsomyndigheten) | https://www.folkhalsomyndigheten.se/ |
|  | The Swedish Agency for Health and Care Services Analysis (Vardanalys) | http://www.vardanalys.se |
|  | Swedish Agency for Health Technology Assessment and Assessment of Social Services | http://www.sbu.se/en/ |
| **United Kingdom** | Department of Health | https://www.gov.uk/government/organisations/department-of-health |
|  | Genomics England (100 000 Genomes Project) | http://www.genomicsengland.co.uk |
|  | PHG Foundation | http://www.phgfoundation.org/ |
| **EUROPE (GENERAL)** | EuroGentest (European Commission) | http://www.eurogentest.org/index.php?id=160 |
|  | PHGEN (Public Health Genomics European Network) | http://www.phgen.eu/typo3/index.php |
|  | ESHG (European Society of Human Genetics) | https://www.eshg.org/home.0.html |
