# Supplementary material for: Identification of Delivery Models for the Provision of Predictive Genetic Testing in Europe: Protocol for a Multicentre Qualitative Study and a Systematic Review of the Literature
Source: Front Public Health. 2017 Aug 22;5:223. doi: 10.3389/fpubh.2017.00223 (PMC5572240; doi:10.3389/fpubh.2017.00223)
Supplement: Supplementary file 2 [file table_2.docx]

| **Table 2 - WEBSITES CONSULTED AS A STARTING POINT FOR IDENTIFICATION OF POLICY DOCUMENTS IN EXTRA-EU COUNTRIES** | | | |
| --- | --- | --- | --- |
| **COUNTRY** | ORGANIZATION | **WEBSITE** | |
| **Australia** | Department of Health | http://www.health.gov.au/ | |
|  | Human Genetics Society of Australasia | https://www.hgsa.org.au | |
|  | Victorian Clinical Genetics Service (VCGS) | http://www.vcgs.org.au/clinical/ | |
|  |  |  |  |
| **Canada** | Health Canada | http://www.hc-sc.gc.ca/index-eng.php | |
|  | Genomics R&D Initiative (Canadian Government) | http://grdi-irdg.collaboration.gc.ca/eng/genome_canada/index.html | |
|  | GenomeCanada | http://www.genomecanada.ca | |
|  | McGill University Health Centre | https://muhc.ca |  |
|  |  |  |  |
| **New Zealand** | Ministry of Health | http://www.health.govt.nz/ | |
|  |  |  |  |
| **United States** | Department of Health and Human Services (HHS) | http://www.hhs.gov/ | |
|  | CDC | http://www.cdc.gov/genomics | |
|  | National Human Genome Research Institute (part of NIH) | https://www.genome.gov/health/ | |
|  | Genetic Services Policy Project | http://depts.washington.edu/genpol/ | |
|  | PMC (Personalized Medicine Coalition) | http://www.personalizedmedicinecoalition.org | |
|  | Michigan Department of Health and Human Services | http://www.michigan.gov/mdch/ | |
