# Supplementary material for: Identification of Delivery Models for the Provision of Predictive Genetic Testing in Europe: Protocol for a Multicentre Qualitative Study and a Systematic Review of the Literature
Source: Front Public Health. 2017 Aug 22;5:223. doi: 10.3389/fpubh.2017.00223 (PMC5572240; doi:10.3389/fpubh.2017.00223)
Supplement: Supplementary file 3 [file table_3.doc]

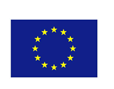

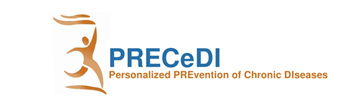

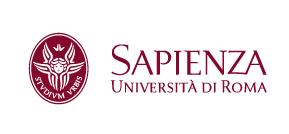


Table 3 - DATA EXTRACTION FORM FOR SCIENTIFIC ARTICLES ON GENETIC SERVICE DELIVERY MODELS

# PART 1. GENERAL DESCRIPTION OF THE STUDY AND THE GENETIC SERVICE

| **SECTION** | **RELEVANT INFORMATION TO EXTRACT** | |
| --- | --- | --- |
| **1.1 Study description** | **Author, year of publication (first author of the study and pubblication year):** | |
|  | **Title of the study:** | |
|  | **Country and/or region where the genetic service is implemented:** | |
|  | **General description of the genetic service, including the aim of the study:** | |
| **1.2 General context of the Genetic Service** | **Pilot study or integrated regional/national service:**  □ Pilot study  □ Integrated service in the healthcare system  □ Direct-to-Consumer (DTC) service | |
|  | **Year of implementation of the genetic service:** | |
|  | **Type of healthcare system in the country where the service is implemented:**  □ Beveridge Model (National Health System)  □ Bismarck Model  □ National Health Insurance Model (mixed model) | |
|  | **Existence of national or regional policies on genetic services (list the documents):**  □ National plan on public health genomics  □National/regional guidelines on genetic services  □ Other  □ Not reported | |
|  | **Practice setting:**  □ Private  □ Public (specify academic or community based service)………………………………………… | |
|  | **Financing mechanism (the main source of funding for the genetic service):**  □ Private  □ Public  □ Both  □ Not reported | |
|  | **Information dissemination to healthcare providers (means of informing community healthcare providers about the genetic service):**  □ Professional boards  □ Scientific journals  □ Meetings  □ Others (specify……………………………………………………………………………………..)  □ Not reported | |
|  | **Formal training offered to healthcare providers (means used by the genetic service to train providers in genomics):**  □ Formal/continuing education programs  □ Seminars  □ Others (specify………………………………………………………………………………………)  □ Not reported  **Specify the category of healthcare providers that are addressed:** | |
|  | **Information dissemination to patients (means of informing patients about the genetic service):**  □ Service web-site  □ Healthcare providers  □ Media (advertisement on radio, TV, journals, etc.)  □ Others (specify: ……………………………………………………………………………………….)  □ Not reported | |
|  | **Information and communications technologies (ICTs)** **for service organization. Use of communication devices (e.g.: cellular phones, computer, satellite systems, etc.) and the associated services for service organization:**  □ Organization of medical records  □ Videoconferencing  □ Distance learning  □ Others (specify……………………………………………………………………………………………)  □ Not reported | |
|  | **ICTs for communication. Use of communication devices (e.g.: cellular phones, computer, satellite systems, etc.) and the associated services for communication (e.g.: telemedicine, text messaging, etc.)] between:**  □ Genetic service providers and patients  □ Genetic service providers and community healthcare providers  □ Not reported | |
|  | |  |

## PART 2. INFORMATION ON PATIENTS AND PATHWAYS TO CARE

| **SECTION** | **RELEVANT INFORMATION TO EXTRACT** |
| --- | --- |
| **2.1 Patients and pathways to care** | **Target population (the intended users of the service):**  **1. Type of population**  □ Pediatric population  □ Adult population  □ Both  □ Other (specify………………………………….)  **2. Gender**  □ Male  □ Female  □ Both  **3. Specific ages** (specify…………………………………………………………..)  **4. Ethnic group** (specify…………………………………………………………..) |
|  | **Access/referrals to the genetic service. Patients are referred to the genetic service from**:  □ Primary care physicians (GPs)  □ Other medical specialists (specify: ………………………………………….)  □ Public health screening programs. Indicate type of screening carried out (e.g. newborn, breast cancer, colon cancer, etc.)……………………………………………………..  □Direct access to the genetic service/self-referral  □ Other (……………………………………………………………………………) |
|  | **Risk assessment/pedigree data collection:**  **1. Who is responsible**  □ Medical geneticist  □ Genetic counsellor  □ Medical specialist  □ GP  □ Other trained professionals (specify, e.g. genetic nurse, midwife, etc.)…………………………………..  **□** Not provided  **□** Not reported  **2. When is it performed**  □ Prior to counselling  □ During counselling  □ Prior to examination by a physician  □ During examination by a physician  **□** Not reported  □ Other (specify……………………………………………………………………………………)  **3. Which tools are used**  □ Questionnaires  □ Devices  □ Not reported  □ Other (specify……………………………………………………………………………………) |
|  | **Provision of genetic counselling (pre-test consultation):**  **1. Who is responsible**  □ Medical geneticist  □ Genetic counsellor  □ Medical specialist  □ GP  □ Not reported  □ Other (specify………………………………………………………………………………………..)  **2. Consultation time (duration of the consult):** ………………………………………… |
|  | **Provision of genetic testing:**  **1. Specify the type of genetic tests offered:** ……………………………………………………….  **2. A consent form is obtained before testing:**  □ Yes  □ No  □ Not reported  **3. Who is responsible for test requesting**  □ Medical geneticist  □ Genetic counsellor  □ Medical specialist (specify, e.g. cardiologists, neurologists, endocrinologists, etc.)  □ GP  □ Other (specify…………………………………………………………………………………………………….)  □ Not reported  **4. Cascade testing is considered (testing offered to relatives of individuals at risk)**  □ Yes  □ No  **□** Not reported  **5. How are relatives contacted**  □ The genetic service asks the proband for permission to contact relatives directly  □ The genetic service asks the proband for permission to contact relatives via a physician  □ The genetic service asks patients to suggest to their relatives that they should undergo testing  □ Other (specify……………………………………………………………………………………………..)  □ Not reported |
|  | **Provision of genetic counselling: post-test consultation**  **1. Who is responsible**  □ Medical geneticist  □ Genetic counsellor  □ Medical specialist (specify, e.g. cardiologists, neurologists, endocrinologists, etc.)  □ GP  □ Other (specify…………………………………………………………………………………………………….)  □ Not reported  **2.** **Consultation time** **(duration of the consult):** …………………………………………………………………………. |
|  | **Post-clinic letter to referring doctor:**  □ It is routinely send  □ It is rarely send  □ It’s never send  □ Not reported |
|  | **Follow-up:**  **1. Follow-up services are provided to patients (specify recommendations and duration)**  □ Yes  □ No  **□** Not reported  **2. Duration of the follow up period:** ………………………………………………. |
| **2.2 Evidence of efficacy and effectiveness** | **Guidelines and recommendations of scientific societies:**  □ Evidence of efficacy and effectiveness are reported in the study  □ Evidence of efficacy and effectiveness fall in one of the Tiers of the CDC recommendation* (Specify the tier……………………………………………………………………..) |
| **2.3 Evidence of cost-effectiveness** | **Guidelines and recommendations of scientific societies:**  □ Cost-effectiveness of the intervention is reported and derives from the present study  □ Cost-effectiveness of the intervention is reported and derives from other studies  □ Cost-effectiveness of the intervention is not reported in the present study |
| **2.4 Feasibility analysis**** | **A feasibility analysis and evaluation of the proposed project was performed:**  □ Yes  □ No |

*Genomic and family health history applications which have a base of synthesized evidence supporting implementation into practice. Center for Disease Control and Prevention: Genetic Testing. Available at: http://www.cdc.gov/genomics/gtesting/tier.htm

**An analysis and evaluation of a proposed project to determine if it is technically and economically feasible.

## PART 3. GENETIC SERVICE EVALUATION

| **SECTION** | **RELEVANT INFORMATION TO EXTRACT** |
| --- | --- |
| **3.1 Genetic Services Delivery Model** | **Try to identify the described genetic services delivery model and the associated pathway(s) among the following or any other additional category:**  □ Model I: Genetic services led by geneticists  Patients’ Pathways:  a) Patient - GP/medical specialist - Counsellor - Lab  b) Patient - Counsellor – Lab  □ Model II: Primary Care Model  Patients’ Pathways:  a) Patient - GP - Counsellor - Lab  b) Patient - GP - Lab.  □ Model III: Medical Specialist Model  Patients’ Pathways:  a) Patient - Medical specialist - Lab  b) Patient - Medical specialist - Counsellor - Lab  □ Model IV: Genetic services integrated into population screening programs  Patients’ Pathways:  a) Patient - Doctor - Counsellor - Lab  b) Patient - Doctor - Lab  c) Patient - Counsellor - Lab  □ Model V: Direct to consumer (DTC)  Patients’ Pathways:  Patient – Lab  □ Other (specify…………………………………………………………………………………………….) |
| **3.3 Genetic Service capacity and evaluation** | **Geographic areas and population served (indicate one):**  □ Urban/metropolis □ Rural □ Both (urban and rural)  □ Local □ Regional □ National |
|  | **Staff with specific background in genetics:**  □ Physicians (specify……………………………………………………………………..)  □ Nurses (specify…………………………………………………………………………..)  □ Counsellors  □ Lab staff (specify………………………………………………………………………..)  □ Other (specify…………………………………………………………………………….) |
|  | **Staff without specific background in genetics but with other specialties (specify):**  □ Physicians (specify……………………………………………………………………..)  □ Nurses (specify…………………………………………………………………………..)  □ Counsellors  □ Lab staff (specify………………………………………………………………………..)  □ Other (specify…………………………………………………………………………….) |
|  | **Characteristics of the laboratories**  **1. Quality standards corresponding to regional/national regulations:**  □ Yes  □ No  **□** Not reported  **2. How are the laboratories organized:**  □ Associated with local genetic services  □ Associated with regional genetic services  □ Associated with universities/academic centers  □ Associated with other research facilities (specify………………………………………………………)  □ Private laboratories  □ Other (specify…………………………………………………………………………….)  □ Not reported  **3. Number of laboratories**:…………………………………………………………… |
|  | **Outcome of care: indicators used to evaluate the impact of the service on population’s health**  **1. Process indicators are reported**  □ Yes  □ No    **2.** **Outcome indicators are reported**  □ Yes  □ No |
| **3.4 Issues and strengths of the delivery model** | **1. Critical issues (critical aspects of the genetic service delivery model, barriers to its implementation) are reported:**  □ Yes (specify…………………………………………………………………………….)  □ No  **2.** **Strengths** **(positive aspect of the genetic delivery model that can also facilitate its implementation) are reported:**  □ Yes (specify…………………………………………………………………………….)  □ No |
| **3.5 Conclusions** | **Authors’ comments, recommendations (proposals for the improvement and correct implementation of the genetic service):** |
